# Development of Hybrid *Pleurotus cystidiosus* Strains with Enhanced Functional Properties

**DOI:** 10.3390/foods14244329

**Published:** 2025-12-16

**Authors:** Sung-I Woo, Minji Oh, Hak Hyun Lee, Inseo Song, Se Jeong Kim, Youn-Lee Oh, Ji-Hoon Im, Eun-Ji Lee, Mi Kyeong Lee

**Affiliations:** 1 Mushroom Research Division, National Institute of Horticultural and Herbal Science, Rural Development Administration, Eumseong 27709, Republic of Korea; woosungi1013@korea.kr (S.-I.W.); minji1228@korea.kr (M.O.); o5ne2@korea.kr (Y.-L.O.); jihooni24@korea.kr (J.-H.I.); ejg1105@korea.kr (E.-J.L.); 2 College of Pharmacy, Chungbuk National University, Cheongju 28160, Republic of Korea; leehakhyun1997@cbnu.ac.kr (H.H.L.); psisq0136@chungbuk.ac.kr (I.S.); sejeong@chungbuk.ac.kr (S.J.K.)

**Keywords:** oyster mushroom, polysaccharide, phenolic compounds, ergothioneine, antioxidant activity, α-glucosidase inhibition

## Abstract

This study aimed to develop and evaluate new hybrid strains of *Pleurotus cystidiosus* with enhanced functional and physiological characteristics. Hybridization between the parental strains KMCC01257 and KMCC05164 yielded four promising hybrid lines (PA-054, PA-104, PA-122, and PA-132), which were selected based on superior mycelial growth and yield performance. Morphological traits and productivity were evaluated across three developmental stages: primordium formation (C1), fruiting body development (C2), and maturation (C3). As cultivation progressed, the number of fruiting bodies decreased, whereas total yield per cultivation bag increased, indicating that late-stage management plays a critical role in maximizing productivity. Chemical analyses revealed that water extracts contained higher levels of polysaccharides, ergothioneine, and total phenolics than EtOAc extracts. Among the tested strains, PA-132 exhibited the highest phenolic content and strongest antioxidant activity, while PA-104 showed greater polysaccharide and ergothioneine accumulation than the parental strains. Antioxidant activity increased over developmental stages and was consistently higher in water extracts, whereas α-glucosidase inhibitory activity was detected primarily in EtOAc extracts with minimal variation among strains or stages. Overall, the results demonstrate that hybridization effectively enhanced the biosynthesis of bioactive metabolites and the functional properties of *P. cystidiosus*. The selected hybrid strains, particularly PA-132 and PA-054, represent promising candidates for the development of high-value functional mushroom cultivars and nutraceutical applications.

## 1. Introduction

Edible mushrooms are well known not only as nutritious foods but also as rich sources of bioactive compounds that exhibit diverse physiological functions, including antioxidant, anti-diabetic and immunomodulatory activities [1,2,3]. Among these bioactive metabolites, polysaccharides, phenolic compounds and ergothioneine have attracted particular attention due to their beneficial biological activities [4,5,6]. Mushroom polysaccharides are well known for their broad pharmacological activities, such as immune modulation and regulating glucose metabolism [7,8,9]. Ergothioneine, a sulfur-containing antioxidant amino acid derivative biosynthesized exclusively by fungi and certain bacteria, plays an essential role in protecting cells from oxidative stress and metabolic dysfunction [10,11]. In addition, phenolic compounds contribute significantly to the antioxidant potential of mushrooms [12,13].

The genus *Pleurotus* (oyster mushrooms) is among the most widely cultivated edible mushrooms worldwide because of its rapid growth, efficient utilization of lignocellulosic agro-residues, and favorable nutritional and functional qualities [14,15,16]. Species such as *P. ostreatus*, *P. eryngii*, and *P. cystidiosus* are valued for their protein content, β-glucans, and phenolic antioxidants [17]. *P. cystidiosus*, commonly known as the abalone mushroom, possesses a distinctive flavor, texture, and bioactive profile; however, unlike other *Pleurotus* species, it exhibits substantially slower mycelial growth, higher sensitivity to environmental fluctuations, and lacks standardized cultivation protocols, which together limit its large-scale commercial production [18]. Moreover, information on its metabolite composition, particularly stage-specific variation in polysaccharides, phenolics, and ergothioneine, remains limited compared with other well-studied *Pleurotus* spp.

In mushroom breeding, monokaryon–monokaryon (Mon–Mon) mating is widely used to generate genetically diverse dikaryons. This approach increases crossing efficiency and genetic variability by combining compatible monokaryotic strains [19]. Crossbreeding within *Pleurotus* species has been used to improve mycelial growth, yield, environmental resilience, and metabolite profiles. A few hybrid strains of *Pleurotus* spp. have shown enhanced polysaccharide production or antioxidant capacity relative to their parental lines [20,21,22]. However, no prior studies have systematically characterized hybrid strains of *P. cystidiosus*, and there are no reports comparing developmental-stage-specific variation in polysaccharides, phenolics, or ergothioneine in hybrid strains. Furthermore, although antioxidant and α-glucosidase activities have been examined in some *Pleurotus* species [23,24], integrated analyses linking metabolite profiles to functional activities across defined growth stages are lacking for *P. cystidiosus*.

Therefore, the present study aimed to develop novel hybrid strains derived from the parental strain KMCC01257 and the protoplast-fusion strain KMCC05164 and to test the hypothesis that crossbreeding would (i) improve mycelial growth and yield and (ii) alter the accumulation patterns of key bioactive metabolites, including polysaccharides, phenolics, and ergothioneine, thereby enhancing antioxidant and α-glucosidase inhibitory activities. By integrating morphological, biochemical, and functional evaluations across three defined cultivation stages, this study provides new insights into the metabolic effects of hybridization and contributes to the development of high-value *Pleurotus* strains with improved functional potential.

## 2. Materials and Methods

### 2.1. Fungal Strains

**Figure 1:**
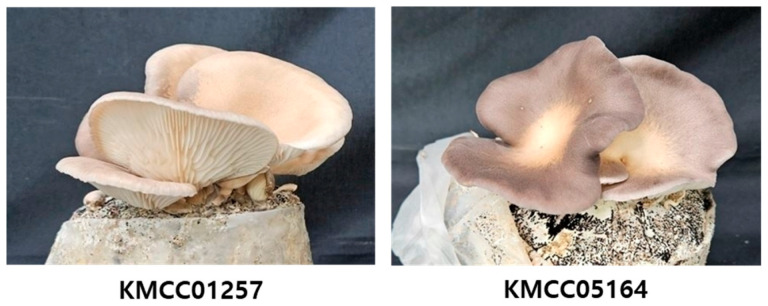
Morphology of fruiting bodies of the parental strain *P. cystidiosus* (KMCC01257) and the protoplast fusion-derived hybrid strain (KMCC05164) grown on bag cultivation.

The fungal strains used in this study were obtained from the Mushroom Research Division, Department of Herbal Crop Research, National Institute of Horticultural and Herbal Science (NIHHS, RDA, Korea). The strains *P. cystidiosus* (accession No. KMCC01257) and KMCC05164 were used as parental strains. The KMCC05164 strain was developed through interspecific protoplast fusion between *P. cystidiosus* ‘Jeonbok-neutari 1-ho’ (KMCC01257) and *Pleurotus ostreatus* ‘Suhan’ (KMCC01680) [25] (Figure 1).

### 2.2. Monospore Isolation and Mating

Basidiospores of *P. cystidiosus* (KMCC01257) and the fusion-derived strain (KMCC05164) were collected from mature fruiting bodies. For monosporic isolation, two sterile toothpicks (2 mm in diameter) were placed in parallel on a sterile Petri dish, and the pileus caps were excised and positioned on the toothpicks to allow spores to be released onto the plate surface. After spore deposition, 100 μL of sterile water was added to suspend the spores. The resulting spore suspension was adjusted to approximately 10^3^–10^5^ spores/mL and spread onto potato dextrose agar (PDA) plates in triplicate. Plates were incubated at 25 °C to induce germination and obtain monokaryotic isolates [26]. A total of 80 spores were isolated from each parental strain, KMCC01257 and KMCC05164.

Mating was performed by inoculating two compatible monokaryotic isolates approximately 20–25 mm apart on the same PDA plate, followed by incubation at 25 °C for about 14 days [27]. Mycelia from the interaction zone were examined under a Nikon E800 microscope (Tokyo, Japan) to confirm the presence of clamp connections, indicating successful dikaryon formation [28]. Microscopic examination revealed that 17 monokaryotic isolates were obtained from KMCC01257 and 8 from KMCC05164, from which 45 dikaryotic strains showing clamp connections were identified. The confirmed dikaryotic strains were subsequently cultured on PDA medium at 25 °C for 20 days.

### 2.3. Substrate Composition

Potato dextrose agar (PDA) medium was prepared and used for the initial inoculation and pre-cultivation of each strain for 20 days. For the preparation of the sawdust substrate, poplar sawdust, beet pulp, and wheat bran were mixed at a mass ratio (*w*/*w*) of 5:3:2. The substrate was further adjusted to a volume ratio (*v*/*v*) of 4:4.8:1 to formulate the final medium. Poplar sawdust was used with a particle size of 2–4 mm, while beet pulp and cottonseed meal were incorporated into the substrate as fine particles smaller than 2 mm. The moisture content of the prepared dried sawdust mixture was adjusted to 70% of dried substrate, and 800 mL of the substrate was placed into 60 polypropylene (PP) cultivation bags for each strain at each C1–C3 stage using the same substrate composition. The filled substrates were sterilized at 121 °C for 90 min, cooled to room temperature, and, after inoculation, incubated under dark conditions in the cultivation room [29].

### 2.4. Cultivation and Growth Evaluation

Mushroom cultivation was carried out for 90 days in a cultivation room maintained at 25–30 °C (20 bags for each cultivation). After 50–62 days of dark incubation, an automatic scraping device equipped with a rotating blade was used to scrape the aged mycelial surface to a depth of 5–7 mm to promote fruiting body formation [30]. The substrates were then transferred to a growing room and maintained at a fruiting initiation temperature of 20–23 °C for 7–8 days, with light intensity controlled at 80–120 lux. Subsequently, cultivation was continued at an optimal growth temperature of 23–25 °C for 15–20 days. Throughout the cultivation period, the relative humidity was maintained at 85–90%, and the CO_2_ concentration was controlled at 800–1000 ppm.

The growth characteristics of the fruiting bodies, including pileus diameter, pileus thickness, and stipe diameter, were assessed by counting mushrooms, performing repeated measurements, and calculating mean values, with three repetitions averaged per bag (g/bag). Growth measurements were conducted using a digital caliper (Mitutoyo Digital Caliper, Kawasaki, Japan). The color parameters (L*, a*, b*) of the pileus and stipe were determined right after harvest using a colorimeter (Chroma Meter CR-400 (Konica Minolta, Osaka, Japan)) [31].

### 2.5. Estimate of Heritability

Broad-sense heritability (H^2^ or Hbs) for yield-related traits was estimated following the approach of Singh and Chaudhary [32]. Heritability was calculated as the ratio of genotypic variance (σ^2^g) to phenotypic variance (σ^2^p):

$$H^{2}=\frac{\sigma^{2}g}{\sigma^{2}p}$$

where phenotypic variance is defined as follows:

σ^2^p = σ^2^e + σ^2^g

Genotypic variance (σ^2^g) and error variance (σ^2^e) were derived from the ANOVA mean squares as follows:

$$\sigma^{2}g=\frac{MSg-KSe}{r}$$

σ^2^e = MSe

where MSg and MSe represent the mean squares for genotype and error, respectively, and r is the number of replications. In this study, yield measurements were obtained from 20 cultivation bags per strain, and thus r = 20.

The ANOVA model used for estimating variances was also explicitly defined. Because the primary objective was to compare yield differences among specific strains, genotype was treated as a fixed effect, while replication was treated as a random effect. Variance components obtained from this model were then used to determine Hbs values for each strain.

Interpretation of heritability followed the criteria of Stansfield and Elrod [33]:

low (Hbs < 0.2), moderate (0.2 ≤ Hbs ≤ 0.5), and high (Hbs > 0.5)

### 2.6. Measurement of Polysaccharide Content [34]

Dried fruiting body powder (1 g) was mixed with distilled water at a ratio of 1:20 (*w*/*v*) and heated at 95 °C for 2 h under gentle agitation (approximately 100 rpm). The resulting extract was collected by centrifugation at 8000× *g* for 15 min. The extraction was repeated twice, and supernatants were pooled and concentrated under reduced pressure to ~1/5 volume. For precipitation, 95% ethanol, equivalent to four times the volume of the aqueous supernatant concentrate, was added to the concentrate, mixed, and kept at 4 °C overnight. The precipitate was collected (8000× *g*, 15 min), washed sequentially with 95% ethanol and acetone, and redissolved in distilled water at the concentration of 10 mg/mL. Then, 1.0 mL of each sample was mixed with 1.0 mL of 5% phenol (*v*/*v*) in a test tube, followed by the addition of 5.0 mL of concentrated sulfuric acid to the mixture. Then the test tube was cooled in an ice bath for 2 min before being stored at room temperature for 15 min. Absorbance was measured at 490 nm using a UV–Vis spectrophotometer (Mecasys, Daejeon, Korea). A calibration curve was obtained from glucose standards (0.1 to 1.0 mg/mL), and total polysaccharides were expressed as mg glucose equivalents (GE) per g dry weight (DW) (mg GAE g^−1^ DW) of mushroom sample. Each sample was measured in triplicate and calculated as mean ± SD.

### 2.7. Measurement of Total Phenolic Content

Dried fruiting body powder (0.5 g) was extracted with distilled water or EtOAc at a ratio of 1:20 (*w*/*v*) under gentle agitation (approximately 100 rpm). The resulting extract was collected by centrifugation at 8000× *g* for 15 min. Then, the Folin–Ciocalteu assay was employed for the determination of the total phenolic content [35]. In brief, 55 μL of each sample was added to a 96-well plate, followed by 5 μL of Folin–Ciocalteu’s phenol reagent. The reaction mixture was incubated for 5 min with gentle shaking, and then 40 μL of 7% Na_2_CO_3_ was added to the reaction mixture. The reaction mixture was kept in a dark condition at room temperature for the reaction. After 90 min of incubation, the absorbance was measured at 630 nm with a microplate reader. The total phenolic content in each sample was expressed as gallic acid equivalent (GAE) using gallic acid (0.02 mg/mL to 1.2 mg/mL) as a standard.

### 2.8. Measurement of Ergothioneine Content [36]

Dried fruiting body powder (500 mg) was extracted with distilled water at a ratio of 1:20 (*w*/*v*) under gentle agitation (approximately 100 rpm). The resulting extract was collected by centrifugation at 8000× *g* for 15 min and passed through a 0.45 m PTFE filter. An HPLC system equipped with Waters 600 Q-pumps, a 996-photodiode array detector, and Waters Empower software (Version 3, Waters Corporation, Milford, MA, USA) was used to quantify the concentration of ergothioneine. The sample was separated using a Phenomenex Gemini-NX C_18_ column (5 µm, 110 Å, 10 × 150 mm; Phenomenex Inc., Torrance, CA, USA) with a combination of methanol and water (10:90, *v*/*v*) for isocratic elution with a running period of 60 min and a detection wavelength of 254 nm. A calibration curve standard (0.1 to 1.0 mg/mL) was obtained from ergothioneine standards, and each sample was measured in triplicate and reported as mean ± SD.

### 2.9. Measurement of DPPH Radical Scavenging Activity

The antioxidant activity was evaluated by measuring the free radical scavenging activity using DPPH [37]. In brief, 2 μL of each sample was added to a 96-well plate, followed by 48 μL of methanol. Then, 50 μL of 0.3 mM DPPH solution was added to each well. The mixture was left to stand for 10 min in the dark, after which the absorbance was measured at 550 nm using a microplate reader (Biotek, Winooski, VT, USA). Ascorbic acid was used as a positive control.

Inhibition (%) = [(absorbance of the control − absorbance of solution with samples)/(absorbance of the control − absorbance of blank)] × 100.

### 2.10. Measurement of α-Glucosidase Inhibitory Activity

α-Glucosidase inhibitory activity was evaluated using α-glucosidase (from *Saccharomyces cerevisiae*, 1 U/mL) and p-nitrophenyl α-D-glucopyranoside as the substrate [38]. Test samples were mixed with 80 μL of enzyme buffer and 10 μL of the enzyme solution and incubated at 37 °C for 15 min. Subsequently, 10 μL of *p*-nitrophenyl α-D-glucopyranoside (10 mM in deionized water) was added to initiate the reaction, followed by incubation at 37 °C for 20 min. The amount of *p*-nitrophenol released was quantified by measuring absorbance at 405 nm using a 96-well microplate reader (Biotek, Winooski, VT, USA).

Inhibition (%) = [(absorbance of the control − absorbance of solution with samples)/(absorbance of the control − absorbance of blank)] × 100.

### 2.11. Statistical Analysis

One-way analysis of variance (ANOVA) followed by Duncan’s multiple range test (DMRT) was performed on polysaccharides, ergothioneine, phenolics, and bioactivities (antioxidant, α-glucosidase) using SAS software version 9.1.3 (SAS Institute, Cary, NC, USA). Significant differences were determined by *p* < 0.05.

## 3. Results

### 3.1. Development of Hybrid Strains of P. cystidiosus

**Figure 2:**
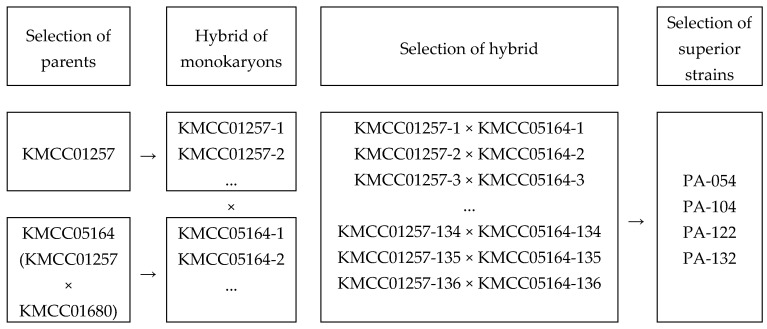
The pedigree of a new cultivar in *P. cystidiosus.*

**Figure 3:**
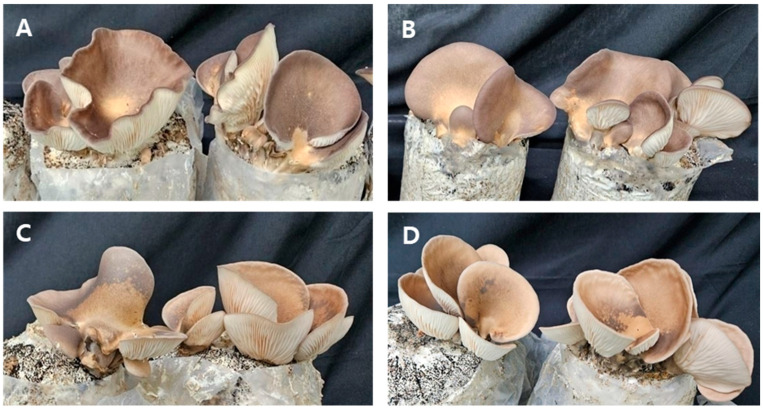
The morphologies of selected *P. cystidiosus* in bag cultivation. (**A**) PA-054, (**B**) PA-104, (**C**) PA-122, (**D**) PA-132.

A total of 80 spores were isolated from each of the *P. cystidiosus* parental strains, KMCC01257 and KMCC05164. Microscopic observation revealed that 17 and 8 monokaryotic isolates, respectively, were obtained from KMCC01257 and KMCC05164. Among them, 45 dikaryotic strains exhibiting clamp connections were confirmed. Based on mycelial growth rate and yield characteristics, four promising strains were selected: PA-054 (KMCC01257-10 × KMCC05164-29), PA-104 (KMCC01257-8 × KMCC05164-41), PA-122 (KMCC01257-10 × KMCC05164-55), and PA-132 (KMCC01257-48 × KMCC05164-55) (Figure 2 and Figure 3).

### 3.2. Cultivation Characteristics

*P. cystidiosus* has a genetic distinction from that of other *Pleurotus* species. Although it is appreciated for its unique aroma and texture, its growth rate is relatively slow, and its development is influenced by substrate composition and environmental cultivation conditions together with genetic factors. Therefore, the cultivation characteristics of the two parental strains and four selected hybrid lines were evaluated under controlled conditions. A mixed substrate consisting of 50% poplar sawdust, 30% beet pulp, and 20% cottonseed meal was packed into 800 mL polypropylene bags and inoculated with each strain. Mycelial growth was completed after approximately 50–62 days at 25–30 °C and 60–70% relative humidity. Primordium formation required 10–11 days at 20–23 °C and 75–80% relative humidity, while fruiting body development was completed within 7–8 days at 23–25 °C and 85–90% relative humidity. These conditions were found to be suitable for bag cultivation of *P. cystidiosus*.

### 3.3. Stage-Specific Growth Characteristics

The morphological and productivity characteristics of six *P. cystidiosus* strains were evaluated at three cultivation stages: primordium formation (C1), fruiting body development (C2), and maturation (C3). Growth characteristics were presented as the mean ± standard deviation (SD) of three harvests per flush for each strain and cultivation stage.

The pileus diameter ranged from 58.1 to 98.6 mm, generally increasing as cultivation progressed. In strain KMCC01257, the pileus diameter increased from 58.5 mm at C1 to 97.9 mm at C3, and a similar trend was observed in KMCC05164 and PA-054. The stipe thickness varied between 10.1 and 22.3 mm, while the stipe length ranged from 8.7 to 34.3 mm, showing moderate variation among strains and stages. PA-132 exhibited the highest individual fruiting body weight (41.8 g) at the C3 stage and achieved the maximum total yield (77.4 g per 800 mL bag) at the C2 stage (Table 1).

The pileus color parameters (L*, a*, b*) also differed significantly between strains and cultivation stages. In PA-132 at the C2 stage, the color values were L* = 39.4 ± 15.7, a* = 5.4 ± 2.0, and b* = 11.7 ± 4.1. The pileus color was measured three times at the center of the pileus, with all measurements conducted within one hour after harvest (Table 2).

Overall, these findings demonstrate that both strain and cultivation stage markedly influence the morphology and productivity of *P. cystidiosus*, highlighting the importance of optimizing stage-specific cultivation conditions to improve fruiting body quality and yield.

### 3.4. Estimation of Heritability on Agronomic Traits

Based on heritability analysis, heritability for yield-related traits was obtained across combined growth stages (Table 3). The results integrated across growth stages for the six strains showed that PA-132 exhibited a high Hbs value of 0.746, while PA-122 showed a moderate Hbs of 0.435. In contrast, KMCC01257 had a low Hbs of 0.024, suggesting that little of the observed phenotypic variance in yield is attributable to genetic variance under these conditions. The remaining strains (KMCC05164, PA-054, and PA-104) exhibited low Hbs values ranging from 0.08 to 0.13. These results indicate that the strain with a high Hbs value is PA-132, while PA-122 exhibits a moderate Hbs, demonstrating their genetic potential for yield.

### 3.5. Total Polysaccharide Content

**Figure 4:**
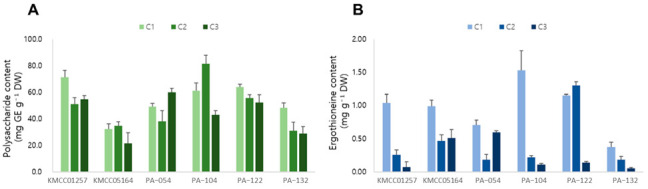
Polysaccharide (**A**) and ergothioneine (**B**) contents of six *P. cystidiosus* strains.

Polysaccharides are key bioactive constituents of mushrooms and are closely associated with various health-promoting properties. In this study, the polysaccharide content of six *P. cystidiosus* strains was quantified across three developmental stages (C1–C3) (Figure 4A).

Overall, polysaccharide levels were highest at the initial primordia-forming stage (C1) and gradually decreased as the fruiting bodies matured (C3). A clear strain-dependent variation was observed. Among the parental strains, KMCC 01257 exhibited a higher polysaccharide content compared with KMCC 05164. Among the newly developed hybrid strains, PA-104 showed the highest polysaccharide content (81.6 ± 6.2 mg GE g^−1^ DW), exceeding that of the parental strain KMCC 01257. Strains PA-054 and PA-122 displayed polysaccharide levels comparable to KMCC 01257, whereas PA-132 exhibited relatively lower levels (28.9 ± 5.1 mg GE g^−1^ DW).

These findings indicate that polysaccharide accumulation varied significantly depending on the genetic background of each strain and developmental stage. The results further suggest that selective hybridization and optimization of harvesting time may enable the development of *P. cystidiosus* strains with enhanced polysaccharide content, potentially improving their functional properties.

### 3.6. Ergothioneine Content

Ergothioneine, a sulfur-containing antioxidant amino acid derivative unique to fungi and certain bacteria, plays an essential role in protecting cells from oxidative damage and maintaining redox balance. Therefore, the ergothioneine contents of water extracts from six *P. cystidiosus* strains were quantified at three cultivation stages (C1–C3) (Figure 4B).

Overall, the ergothioneine levels were significantly higher in the early cultivation stage (C1) except for PA-122 and gradually decreased during fruiting body development. This pattern suggests that ergothioneine biosynthesis is more active during the early stages of primordium formation. Among the strains, the hybrid strain PA-104 exhibited a slightly higher ergothioneine content compared with the parental lines, indicating that hybridization may enhance sulfur-containing metabolite accumulation.

These findings imply that ergothioneine production in *P. cystidiosus* is affected by both developmental stage and genetic background, and that early-stage harvesting may be favorable for maximizing this potent antioxidant compound.

### 3.7. Total Phenolic Content

**Figure 5:**
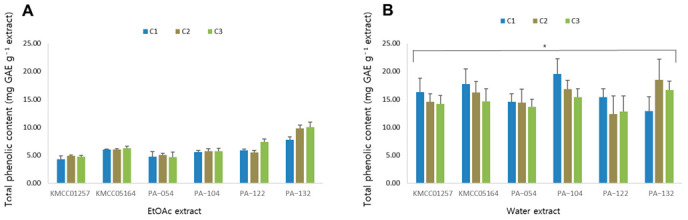
Total phenolic contents of (**A**) EtOAc extract and (**B**) water extract of six *P. cystidiosus* strains. Contents are expressed as mean ± SD of three independent experiments. * significant difference at *p* < 0.05 from the corresponding EtOAc extract.

Phenolic compounds play a crucial role in the antioxidant capacity of mushrooms [37]. Therefore, the total phenolic contents (TPC) of six *P. cystidiosus* strains were determined for both ethyl acetate (EtOAc) (Figure 5A) and water extracts (Figure 5B) at three cultivation stages (C1–C3). Overall, the water extracts exhibited higher phenolic contents (12.8–19.5 mg GAE g^−1^ extract) than the corresponding EtOAc extracts (4.3–10.0 mg GAE g^−1^ extract) in all strains, indicating that phenolic constituents in *P. cystidiosus* are more efficiently extracted with polar solvents.

The hybrid strain PA-132 showed a slightly higher total phenolic content compared with the parental lines, suggesting that hybridization may enhance phenolic content. Regarding cultivation stages, the EtOAc extracts showed the highest phenolic levels at the maturation stage (C3), whereas the water extracts displayed the highest TPC values at the primordium stage (C1).

These results indicate that both genetic background and cultivation stage affect the accumulation of phenolic compounds in *P. cystidiosus* and that the polar solvent is more efficient for the extraction of its phenolic compounds.

### 3.8. Antioxidant Activity

**Figure 6:**
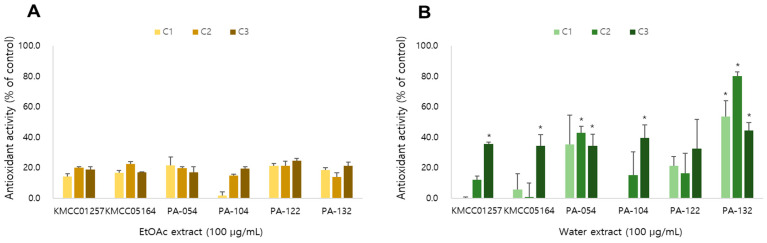
Antioxidant activity of (**A**) EtOAc extract and (**B**) water extract of six *P. cystidiosus* strains. Activity (%) are expressed as mean ± SD of three independent experiments. * significant difference at *p* < 0.05 from the corresponding EtOAc extract.

Antioxidant activity reflects the ability of mushroom-derived metabolites to neutralize reactive oxygen species and protect cellular components from oxidative stress. The antioxidant capacities of EtOAc and water extracts obtained from six *P. cystidiosus* strains were evaluated using DPPH radical scavenging assays (Figure 6).

In general, the water extracts exhibited stronger antioxidant activities than the EtOAc extracts across all strains, suggesting that polar constituents contributed more effectively to the overall activity. The antioxidant potential increased progressively during cultivation, showing higher activity at the later developmental stages compared with the early primordium stage.

Water extract of PA-132 tends to show higher antioxidant capacity, followed by PA-054, both of which exceeded the levels observed in the parental strains. These results indicate that hybridization can enhance the production of antioxidant metabolites in *P. cystidiosus*.

### 3.9. α-Glucosidase Inhibitory Activity

**Figure 7:**
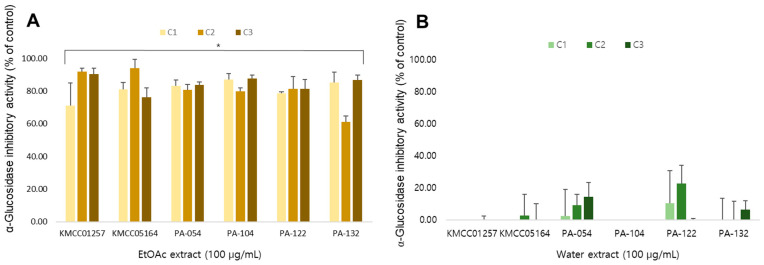
α-Glucosidase inhibitory activity of (**A**) EtOAc extract and (**B**) water extract of six *P. cystidiosus* strains. Inhibition values (%) are expressed as mean ± SD of three independent experiments. Significant difference in the inhibitory activity of EtOAc extract at *p* < 0.05 from the corresponding water extract. Activity (%) are expressed as mean ± SD of three independent experiments. * significant difference at *p* < 0.05 from the corresponding water extract.

The α-glucosidase inhibitory activity was evaluated to assess their potential antidiabetic properties (Figure 7). In contrast to the antioxidant results, EtOAc extracts exhibited markedly higher α-glucosidase inhibitory activity, whereas the water extracts showed no inhibitory effect across all strains and cultivation stages. This pattern may indicate that the α-glucosidase inhibition observed in *P. cystidiosus* is more likely associated with non-polar or moderately polar metabolites, which needs to be confirmed by further study. No significant differences were observed between the six strains or between the cultivation stages, which indicated that, unlike phenolic-driven antioxidant activity, the anti-diabetic potential of *P. cystidiosus* extracts is consistent independent of developmental stages.

## 4. Discussion

This study demonstrated that hybridization among *P. cystidiosus* strains shapes both the morphological and biochemical characteristics of the resulting fruiting bodies. The selected hybrid lines, particularly PA-132 and PA-054, showed improved growth performance, higher yields, and enhanced accumulation of specific bioactive compounds compared with their parental strains. These observations support the potential of crossbreeding within *Pleurotus* species to generate phenotypically diverse lines with functional advantages under defined cultivation conditions.

The heritability analysis further highlighted the breeding potential of these hybrids. PA-132 exhibited the highest broad-sense heritability (Hbs = 0.746), indicating a strong genetic contribution to its yield-related traits, while PA-122 showed a moderate Hbs (0.435). Strains with higher heritability values are expected to respond more effectively to selection, even at moderate selection intensity. Thus, PA-132, and to a lesser extent PA-122, represents a promising candidate for future breeding programs aimed at improving productivity in *P. cystidiosus*. However, these implications remain preliminary given the limited number of hybrids and cultivation environments evaluated.

Distinct patterns of metabolite accumulation were also observed across strains and developmental stages. Total phenolic content (TPC) and antioxidant capacity were consistently higher in water extracts, reflecting the predominance of polar, water-soluble phenolic compounds in determining antioxidant potential. This aligns with previous findings in *P. ostreatus* and *P. eryngii*, where phenolic biosynthesis is often stimulated during early fruiting body development as part of oxidative stress mitigation [39,40,41]. Notably, the TPC values measured in PA-132 fall within, and in some cases exceed, the ranges reported for these related species [42], suggesting that hybridization may enhance phenolic metabolism beyond typical species-level variability.

Polysaccharides and ergothioneine, in contrast, exhibited pronounced stage-dependent and strain-specific variations. Polysaccharides peaked at the primordium stage and declined during maturation, consistent with their roles in structural formation and protection during early tissue differentiation. The highest polysaccharide level, observed in PA-104 (81.6 ± 6.2 mg GE/g DW), was slightly higher than values reported for *P. eryngii* and *P. ostreatus* [43]. Ergothioneine accumulated most strongly in the earliest developmental stages, in line with its role as a sulfur-containing cellular antioxidant. The elevated levels in PA-104 suggest potential hybrid-induced alterations in sulfur metabolism, possibly through enhanced activity of enzymes related to ergothioneine biosynthesis.

Together, these findings indicate that phenolics, polysaccharides, and ergothioneine follow distinct developmental trajectories and respond to different genetic regulatory mechanisms. The contrasting metabolic profiles of PA-132 (phenolic- and antioxidant-rich) and PA-104 (polysaccharide- and ergothioneine-rich) illustrate that hybridization can lead to divergent metabolic specialization rather than uniform enhancement across all traits. Such specialization offers valuable opportunities for targeted strain development depending on the desired functional properties. Nevertheless, elucidating the underlying metabolic pathways will require transcriptomic or targeted metabolomic analyses to determine how hybridization alters the regulation of phenolic, polysaccharide, and sulfur-containing metabolite biosynthesis.

α-Glucosidase inhibitory activity, in contrast to antioxidant capacity, was detected primarily in EtOAc extracts and exhibited minimal variation among strains or developmental stages. This suggests that the responsible compounds are likely non-polar constituents, such as terpenoids or sterols, and points to a different metabolic origin than the polar phenolics driving antioxidant activity.

Despite the strengths of this study, several limitations should be acknowledged. All strains were cultivated under a single environmental condition, and their performance may vary under different substrates, climates, or production systems. Additionally, although over forty hybrids were initially generated, only four were analyzed in detail, limiting generalization across the larger hybrid population. The biochemical activities reported here were based solely on in vitro antioxidant and α-glucosidase assays, and no cellular or in vivo validation was performed. Therefore, the functional implications of the observed metabolite profiles require further confirmation. Recognizing these limitations provides a more balanced interpretation of the findings.

In summary, this study provides evidence that hybridization can generate *P. cystidiosus* strains with distinct phenotypic and biochemical traits, including enhanced phenolic content, antioxidant capacity, polysaccharide levels, and ergothioneine accumulation, depending on the hybrid combination. When considered alongside previous work in other *Pleurotus* species, the results suggest that crossbreeding within the genus may serve as a useful strategy for improving selected agronomic or functional traits. However, broader validation across additional genotypes, environments, and mechanistic analyses will be essential before definitive conclusions about breeding potential can be drawn. Continued integration of hybridization, multi-environment testing, and pathway-level analyses will aid in developing high-value mushroom strains suited for functional foods, dietary supplements, and nutraceutical applications.

## 5. Conclusions

In this study, hybrid strains of *P. cystidiosus* were generated through crossbreeding between the parental lines KMCC01257 and KMCC05164, and their morphological characteristics, metabolite profiles, and in vitro functional properties were systematically evaluated under a single cultivation condition. Rather than showing uniform superiority, the hybrids exhibited strain-specific advantages compared with the parental strains. PA-132 demonstrated comparatively higher phenolic content and antioxidant activity, whereas PA-104 displayed elevated polysaccharide and ergothioneine levels, reflecting distinct metabolic specializations that may arise from hybridization. These findings suggest that genetic recombination can diversify biosynthetic capacities within *P. cystidiosus*, although confirmation across broader hybrid populations and cultivation environments will be required.

Overall, the results indicate that hybridization may serve as a useful approach for improving selected agronomic or biochemical traits in mushrooms. The biochemical profiles of PA-132 and PA-104 highlight their potential as promising candidates for further development as functional mushroom strains. Future investigations incorporating multi-environment testing, pathway-level analyses (e.g., transcriptomics or targeted metabolomics), and in vivo validation will be essential to fully assess their commercial and nutritional value and to refine breeding strategies for high-value functional mushroom cultivars.

## Figures and Tables

**Table 1:** Interaction between isolate and stage on the productivity of six *P. cystidiosus* strains in the bag cultivation.

Isolates	Stage	Pileus		Stipe		Productivity	
		Diameter (mm)	Height (mm)	Thickness (mm)	Length (mm)	Individual Weight (g)	Yield (g/800 mL Bag)
KMCC 01257	C1	58.5 ± 0.7 ^c^	58.5 ± 11.8 ^b^	12.9 ± 0.9 ^a^	34.3 ± 44.6 ^a^	19.4 ± 5.1 ^c^	58.2 ± 2.8 ^a^
	C2	72.7 ± 31.2 ^b^	59.1 ± 23.1 ^b^	11.2 ± 5.4 ^b^	8.7 ± 17.5 ^c^	20.2 ± 8.8 ^b^	57.8 ± 25.8 ^a^
	C3	97.9 ± 41.8 ^a^	71.0 ± 19.3 ^a^	10.6 ± 3.8 ^c^	17.5 ± 22.6 ^b^	32.1 ± 9.7 ^a^	53.1 ± 17.7 ^b^
KMCC 05164	C1	76.2 ± 5.1 ^b^	63.0 ± 21.7 ^c^	10.1 ± 2.1 ^b^	16.5 ± 6.0 ^b^	24.8 ± 4.9 ^c^	58.3 ± 11.8 ^b^
	C2	89.3 ± 33.7 ^a^	84.2 ± 25.4 ^a^	19.0 ± 7.3 ^a^	13.9 ± 6.0 ^b^	31.8 ± 10.8 ^b^	48.4 ± 19.1 ^b^
	C3	95.2 ± 26.5 ^a^	77.0 ± 23.6 ^b^	14.9 ± 5.5 ^a^	19.4 ± 7.0 ^a^	36.2 ± 10.6 ^a^	52.5 ± 18.6 ^a^
PA-054	C1	60.9 ± 6.7 ^b^	42.0 ± 25.2 ^b^	22.3 ± 5.5 ^a^	19.3 ± 8.0 ^a^	34.5 ± 3.8 ^a^	54.5 ± 23.6 ^a^
	C2	80.2 ± 2.6 ^a^	55.0 ± 7.8 ^ab^	14.9 ± 3.5 ^b^	11.4 ± 10.1 ^b^	29.5 ± 16.5 ^b^	54.7 ± 21.3 ^a^
	C3	98.6 ± 41.8 ^a^	59.8 ± 23.8 ^a^	14.6 ± 5.8 ^b^	9.3 ± 1.4 ^b^	26.5 ± 6.1 ^b^	64.8 ± 27.8 ^a^
PA-104	C1	59.2 ± 1.7 ^c^	72.3 ± 10.2 ^a^	12.2 ± 6.6 ^c^	15.2 ± 4.1 ^b^	18.8 ± 10.3 ^c^	71.0 ± 11.0 ^a^
	C2	74.9 ± 29.3 ^b^	58.1 ± 22.6 ^c^	14.6 ± 3.8 ^b^	17.6 ± 6.5 ^a^	27.0 ± 8.4 ^a^	57.7 ± 22.2 ^b^
	C3	86.8 ± 25.7 ^a^	68.5 ± 20.5 ^b^	14.9 ± 4.2 ^a^	14.3 ± 5.9 ^c^	25.5 ± 9.2 ^b^	55.1 ± 18.4 ^b^
PA-122	C1	58.9 ± 14.7 ^c^	51.3 ± 10.9 ^c^	14.9 ± 5.3 ^c^	22.6 ± 8.5 ^c^	22.4 ± 9.6 ^c^	42.6 ± 14.1 ^c^
	C2	64.7 ± 23.7 ^b^	57.2 ± 22.1 ^b^	17.9 ± 7.0 ^b^	29.8 ± 12.1 ^b^	27.3 ± 8.8 ^b^	53.8 ± 20.6 ^a^
	C3	80.2 ± 21.2 ^a^	59.1 ± 17.4 ^a^	19.8 ± 6.4 ^a^	29.2 ± 9.2 ^a^	33.8 ± 9.2 ^a^	47.2 ± 16.3 ^b^
PA-132	C1	58.1 ± 3.2 ^c^	60.7 ± 8.7 ^c^	14.8 ± 6.7 ^c^	20.8 ± 17.4 ^c^	30.2 ± 9.9 ^c^	57.2 ± 1.8 ^c^
	C2	62.9 ± 24.1 ^b^	64.6 ± 27.9 ^b^	15.5 ± 4.6 ^b^	28.9 ± 12.7 ^a^	32.1 ± 17.1 ^a^	77.4 ± 1.2 ^a^
	C3	87.9 ± 26.3 ^a^	73.0 ± 23.1 ^a^	18.7 ± 5.1 ^a^	23.3 ± 10.2 ^b^	41.8 ± 12.2 ^a^	66.7 ± 6.4 ^b^

Different letters in the same column indicate means with statistically significant differences (*p* < 0.05). Values are expressed as mean ± SD (*n* = 20 bags).

**Table 2:** Interaction between isolate and stage on the morphologic characteristics.

Isolates	Stage	Pileus Color		
		L*	a*	b*
KMCC 01257	C1	34.6 ± 3.0 ^b^	5.7 ± 0.3 ^c^	8.1 ± 1.1 ^c^
	C2	40.0 ± 16.1 ^a^	6.9 ± 2.9 ^b^	11.1 ± 4.3 ^b^
	C3	36.7 ± 11.1 ^b^	8.0 ± 2.2 ^a^	12.5 ± 3.5 ^a^
KMCC 05164	C1	34.9 ± 9.1 ^c^	4.2 ± 1.0 ^a^	8.5 ± 2.8 ^c^
	C2	47.6 ± 15.8 ^a^	3.5 ± 1.1 ^b^	12.6 ± 4.1 ^a^
	C3	41.3 ± 12.5 ^b^	4.0 ± 1.0 ^ab^	11.5 ± 3.3 ^b^
PA-054	C1	27.7 ± 3.9 ^c^	4.9 ± 0.8 ^c^	5.6 ± 1.5 ^c^
	C2	37.0 ± 16.0 ^a^	5.4 ± 1.3 ^b^	10.2 ± 5.1 ^a^
	C3	35.1 ± 9.0 ^b^	6.5 ± 2.3 ^a^	10.3 ± 2.7 ^a^
PA-104	C1	45.8 ± 9.5 ^a^	6.3 ± 0.8 ^a^	11.3 ± 2.8 ^a^
	C2	36.6 ± 13.8 ^c^	5.6 ± 2.0 ^b^	8.1 ± 3.3 ^c^
	C3	41.0 ± 11.6 ^b^	6.5 ± 1.9 ^a^	10.9 ± 3.3 ^b^
PA-122	C1	31.8 ± 9.9 ^b^	4.4 ± 2.3 ^b^	8.3 ± 3.9 ^b^
	C2	39.1 ± 17.0 ^a^	4.6 ± 2.6 ^a^	10.5 ± 5.5 ^a^
	C3	28.1 ± 12.8 ^c^	3.1 ± 1.7 ^c^	6.5 ± 4.2 ^c^
PA-132	C1	34.3 ± 6.9 ^c^	5.7 ± 1.6 ^a^	10.3 ± 2.6 ^b^
	C2	39.4 ± 15.7 ^a^	5.4 ± 2.0 ^ab^	11.7 ± 4.1 ^a^
	C3	37.7 ± 12.9 ^b^	5.0 ± 1.6 ^b^	11.8 ± 3.8 ^a^

Values are expressed as mean ± SD (*n* = 20 bags). Different letters in the same column indicate means with statistically significant differences (*p* < 0.05). L*: lightness, a*: redness, b*: yellowness.

**Table 3:** Variance components and heritability estimates of six *P. cystidiosus*.

Isolate	σ^2^e*	σ^2^g	σ^2^p	Hbs	Criteria Hbs
KMCC 01257	271.5	6.42	277.92	0.024	Very Low
KMCC 05164	70.09	6.07	76.16	0.080	Low
PA-054	380.23	34.29	414.52	0.083	Low
PA-104	323.88	49.31	373.19	0.132	Low
PA-122	146.03	112.42	258.45	0.435	Moderate
PA-132	14.83	43.76	58.59	0.746	High

σ^2^e*: error variance, σ^2^g: genotypic variance, σ^2^p: phenotypic variance, Hbs: broad sense heritability, Criteria Hbs: The standard for heritability values is low (h^2^ bs < 0.2), moderate (0.2 ≤ h^2^ bs ≤ 0.5), or high (h^2^ bs > 0.5).

## Data Availability

The original contributions presented in the study are included in the article. Further inquiries can be directed to the corresponding author.

## Abbreviations

The following abbreviations are used in this manuscript:

EtOAc	Ethyl acetate
PDA	Potato dextrose agar
